# Ricord on the Treatment of Syphilis

**Published:** 1872-12

**Authors:** 


Ricord On the Treatment of Syphilis.—At the last meeting of the British Medical Association, at Birmingham, last August, Ricord, as a guest of the Association, delivered a lecture on the treatment of syphilis, which, according to the *Lancet* of August, contains the following principal points: As soon as the existence of hard chancre, with indolent swelling of the lymphatic glands, is ascertained to exist, mercurial treatment must be at once adopted to prevent the occurrence of secondary symptoms. This treatment must, according to his experience of forty years, be continued, not until these symptoms have disappeared, but for a period of from five to six months, and must be followed by the use of iodide of potash, if any return of the disease manifests itself during that time. Patients, unless they consent to this long continuation of treatment as the only means to accomplish a radical cure, are not taken by him. Complications with scrofulosis, scurvy, cutaneous diseases, etc., make a cure much more difficult, and general constitutional treatment must, in such cases, precede the anti-syphilitic treatment. Amongst the mercurials, Ricord considers the protojoduret of mercury the most efficient. Where the stomach does not tolerate this remedy, he substitutes mercurial friction as alike efficient, but more disagreeable, unless eruptions, such as eczema, etc., forbid their use. The iodide of potash must be given in large doses of from forty to one hundred grains per day, as this agent is quickly eliminated, and acts, figuratively speaking, by sweeping the blood. The remedy intended to combat tertiary symptoms must be combined with the mercury, whenever a complication of secondary and tertiary symptoms exist together. A return to the mercurial treatment, if the iodide of potash fails to act promptly, very often renders the very best service; frequently, however, when the system has been exposed a long time to the effects of syphilis, an abandonment of all specific treatment, and restitution of the constitution by general tonic treatment becomes essentially necessary.

Bromide of potash, which has lately been recommended as an anti-syphilitic remedy, is not considered as such by him, while it is undoubtedly a valuable aid in suppressing the nervous manifestations of the disease. Certain consequences of syphilis, for instance, necrosis of the bones, are not benefited by specific treatment. A general surgical treatment has to be instituted in such cases. He is firmly convinced that syphilis can be cured, radically, if patients as well as physicians possess the necessary patience and endurance. He does not believe in the critical value of salivation, but considers it of great importance to guard against it as carefully as possible. Only in syphilitic iritis he has frequently seen an improvement of the inflammation with the commencement of salivation.—*Berliner Klinische Wochenschrift, No.* 40, 1872.

